# Covid-19 and the Epigenetics of Learning

**DOI:** 10.1007/s42438-020-00190-9

**Published:** 2020-09-25

**Authors:** Mark William Johnson, Elizabeth Maitland, John Torday

**Affiliations:** 1 University of Liverpool, Liverpool, UK; 2 University of California, Los Angeles, USA

**Keywords:** Covid-19, Epigenetics, Evolutionary biology, Intellectual depth, Institutional organisation

## Abstract

Covid-19 is a natural phenomenon that has rapidly upended much of the cultural infrastructure of societies across the globe. Education, which in recent years increasingly tied itself to notions of global culture and markets, is deeply threatened by these changes to the natural environment. This paper makes the case that the relationship between nature and culture in education requires a deep level analysis of the biological and physical substrate of human learning. Only with a sufficiently fundamental level of analysis can society reorganise its systems of learning and scientific inquiry to this rapidly changing environment. Drawing on evolutionary biology, we argue that institutional and individual structures and processes are recapitulations of evolutionary cellular development. Understanding the impact of Covid-19 on cells presents an invitation to consider the larger-scale cultural recapitulations of similar mechanisms and structures, and this has implications for the ways education might most effectively deploy technology. Whilst universities seek to maintain their existing structures, practices and business models, a cellular evolutionary approach points to the necessity for fundamental rethinking of intellectual life and learning. We consider the parameters of effective educational organisation in a post-Covid-19 world. As the richness and variety of the physical campus is removed, viable educational relationships will necessitate deeper intellectual connections and personal inquiries than are currently permitted in the transactional processes of education.

## Introduction: Learning, Cells and Technology

Learning begins with cells and leads to minds, ideas and universities. As Simon Conway Morris noted, ‘[f]irst there were bacteria, now there is New York’ (Morris 2011). A fundamental question is, what is the connection between cellular mechanisms, social structures and human behaviour? The Covid-19 virus has created an opportunity for revisiting the project first addressed by Piaget (1970), and later by Papert (1991) that asked about the connection between the environment and biological, psychological and behaviourial processes of learning, and how the social organisation of education might address this. What light can be shed on this by understanding the effects of Covid-19 on biological, psychological and sociological phenomena? In addressing these questions, this paper explores the possibilities for research opened-up by recent advances in biology, and particularly the science of epigenetics (Moore 2015; Torday 2020). We argue that a renewed focus on biological mechanisms that relate organic development to environment, and further provide possible integrations between biology and higher-order functions (mind, education and society) presents new empirical approaches to understand education’s relationship to organic development, evolution, history and the environment. In suggesting a unified approach to learning and epigenetic mechanisms, we present the parameters that are revealed as critical steps towards transforming educational practice and technology in the wake of the virus.

Viruses affect cells in ways which interrupt or even destroy higher-level functions. Covid-19 lays bare the extent to which mechanisms of cellular self-organisation are connected to higher-order functions which on the face of it appear distant from biology (for example, political economy). Whilst this is not to suggest a reduction of all activity to biological processes, it is to suggest that systemic multi-layer inter-relationships between biological processes and human organisation are now exposed and require new theory and further study. The speed and scale of destruction and transformation in our higher-order processes of education, economies and the social fabric produced by Covid-19 are arguably beyond anything seen for over 100 years. Understanding how to adapt our processes of learning in the light of the response to the spread of the virus demands a deeper understanding of the relationship between cellular processes and the organisational processes of human communication—much of which is now conducted through digital technology.

Educational institutions are impacted by the natural environment every day: it is partly why managing the estate of the university is so complex and expensive. Being forced to close the campus estate and rely on online tools instead raises further questions about the differences between the cellular processes operating in a face-to-face environment, and the cellular processes operating when we learn, interact and process information online. In the shift to an online learning environment, our central argument is that there has been a consequent shift from one epigenetic environment—that of the physical campus, with its libraries, lecture theatres, coffee bars and pubs—to another epigenetic environment, typically that of the ‘home’. Moreover, there has been a shift from face-to-face communication to communication conducted online, some of it producing high levels of stress for both teachers and learners. This environment of increased stress carries educational and evolutionary risks which demand careful analysis and empirical investigation.

Epigenetics concerns cellular processes of interaction between DNA and epigenetic ‘marks’, where an organism’s patterns of behaviour leave marks in the form of proteins and chemicals in the environment, which are picked-up by other organisms sharing the same environment. As a science, it brings empirical focus to the interaction between organism, species and environment. In drawing attention to the role of epigenetics in learning and education in this paper, we highlight how recent research work in biology has explored this relationship, and what import this carries for our understanding of learning in a transformed environment.

The paper is divided into two parts. In part 1, the background to the elaboration of an approach to epigenetic theory which unites nature with culture through evolutionary biology is expounded. Part 2 explores the relevance of this for understanding human development, learning and their institutional conditions. For some readers, the arguments in the paper may evoke critical responses. To address these, the final section is a critical reflection which engages with three potential areas of objection to our arguments.

## Part 1: Evolution, Nature and Culture

### Epigenetics and the Biology of Learning

Epigenetics is the study of the interaction between environmental conditions and organic development. Rather than view the development of an organism as the total result of heritable genes which determine the development of phenotypes (the observable traits and characteristics of the organism), epigenetics sees phenotypical development as heritable through the deposit and absorption (endogenisation) of environmental ‘marks’—proteins and chemicals produced through organic development—which can be instrumental in transforming the expression of proteins from DNA from one generation to another. Consequently, against the prevailing interpretation of Darwinian theory, acquired characteristics can be inherited through environmental transformations associated with behaviour. Whilst there is a large body of empirical evidence to uphold this view, the full implications of it on education and social policy more generally are as yet under-explored.

The epigenetic approach can, for example, provide deeper explanatory frameworks for established theories and evidence from psychology. To illustrate, Bowlby’s attachment theory (Bowlby 1997; Bowlby 1988), which has long been considered an important component in theories about child development, has recently been explored for an epigenetic connection. This enriches Bowlby’s idea that ‘proximity’ between the child and care-giver is the critical variable in attachment, through an empirical search for epigenetic marks within the attachment relationship (Pietromonaco and Powers 2015; Robakis et al. 2020; Ein-Dor et al. 2018).

Beyond psychological evidence, there is evidence for the root causes of diseases like asthma having epigenetic roots. In studies of the relationship between cigarette smoking and asthma, a quantitative relationship between smoking behaviour of grandparents, parents and asthma in children exists. The process of cellular endogenisation and endosymbiosis—the cellular absorption of epigenetic marks from the environment—first identified by Margulis (1998) shows that the epigenetic marks of smoking are endogenised in the zygote of the childrens’ offspring (the grandchildren). By this mechanism, cellular development carries an ongoing memory of its own history under various conditions of environmental stress. Further evidence exists that epigenetic marks in the form of hormones like oxytocin, cortisol and androgens shape behaviour at different stages of human development. For example, biological development in youth expresses androgens producing a fight-or-flight mechanism, whilst in older age, in lieu of waning androgens, the production of oxytocin produces more altruistic behaviour (Torday 2020). Echoing Haekel’s ‘biogenetic law’ (Torday 2013), these epigenetic processes involve both the developmental process of the individual organism (ontogeny), and the ongoing development of the species (phylogeny), whereby ontogeny can be seen to recapitulate phylogeny.

Epigenetics presents an empirically testable paradigm whereby environment and culture become critically entwined factors in human development, thus providing a new lens through which to view older debates about higher learning and knowledge in society—most notably the division between culture and nature that C.P. Snow identified (Snow 2013). This is particularly important when social behaviours suffer major disruption and have to reconstitute themselves under very different environmental (and consequently, epigenetic) conditions. This is, of course, the very situation that has confronted education in the wake of Covid-19.

### Epigenetics, Consciousness and Matter

An explanatory gap exists, however, in understanding the underlying mechanism that connects production of epigenetic marks and the cellular endogenisation of those marks in development, particularly with cognitive development which leads to the reproduction and transformation of social behaviours. These social behaviours then generate further epigenetic marks. Torday and Rehan (2004) have directly addressed the problem of interconnection between the epigenetic processes, cellular endogenisation and conscious/cognitive development through a theory that extends Margulis’s work back to basic mechanisms of cellular communication and the origin of matter. Stressing the importance of cellular communication and organisation in basic processes of maintaining cellular viability through constructing a ‘niche’, Torday argues that complex biological and cognitive functions can be traced back to fundamental primeval cellular origins (Torday 2012). More significantly, consistent with Margulis’s theories, these primeval viable cellular states co-exist within the structures of more complex organisms, from blastula to brains.

In other words, biological systems, such as human beings, or systems which sit on complex biology such as consciousness, contain within them a vector which points back to primeval cellular origins. Indeed, one may push back further. All organisms are made of cells, and cells are made of molecules, atoms, quarks and so on. Individual cells, like larger organisms, must maintain their survival in their environment. Inquiry into the molecular origin of lipids, cholesterol (Deamer 2017), primeval cells and the primitive forms of early cellular communication produced by calcium pumps (Case et al. 2007) points to the origins of matter itself, or a ‘big bang’ at the beginning of the universe (Torday 2012).

The justification for this argument sits on a range of empirical evidence that highlights the ways in which cells reorganise themselves under stress to prior states of evolutionary history. In studies on the impact of emphysema on the cells of the lung, the epithelial cells of the lung resemble the cells of the lung of the frog. In other words, the disease causes stress on the cellular niche within which the lung cells exist causing them to regress to a prior evolutionary state in order to maintain their viability within this new environment (Torday and Rehan 2004).

In studying how cells maintain viability, Torday has noted three fundamental variables in cellular organisation: the maintenance of homeostasis (the tendency to maintain a constant internal environment), the gaining of energy from the environment through a process called ‘chemiosmosis’, and a process of life creating order against the prevailing entropy of the universe—what Schrödinger called ‘neg-entropy’ (Schrödinger 2012). These three processes Torday calls the ‘First Principles of Physiology’, pointing out that the same processes recapitulate at all levels of cellular organisation. Moreover, since the results of cellular organisation also produce epigenetic marks in the environment, the recapitulation of the same processes can be seen to unite the development of the individual organism (ontogeny) with the ongoing development of the species (phylogeny).

Taken to a social level, this means that the epigenetics of the individuals who make up social organisations contribute to the biological substrate upon which the ongoing functioning of those organisations sits. Environmental stresses of all kinds—whether a pandemic, or political turmoil, economic austerity, poverty, unemployment, or war—can disrupt these processes in ways by which epigenetic mechanisms produce heritable marks for future generations. This is naturally particularly pertinent in educational organisations.

### The Natural Environment of Educational Organisations

Epigenetics provides a mechanism for explaining the interconnection between nature and culture, the boundary between which Covid-19’s impact on education has brought into sharp relief. Among the many profound questions this raises in education, the most basic concerns the appropriate level and unit of analysis. Educational researchers represent a discourse that is underpinned by social structures in university departments that are now almost universally threatened. With Covid-19, we have a dramatic change to the natural environment that threatens the cultural environment in a way which highlights the inter-connectedness between the two.

Snow’s complaint that ‘for many educated people, the second law of thermodynamics elicits confusion or even contempt’ (Snow 2013) still stands today. The physics (and biology) of social systems has been rarely discussed since Comte’s original ambition for sociology to be a ‘social physics’ (Iggers 1959). The fundamental distinctions between nature and culture were subsumed in an institutionally promoted educational research discourse that saw ‘culture’ as policy, management, curriculum and technology, and occasionally anthropology, and ‘nature’ in the methods of psychology, the metrics of learning (although these have typically served institutional not individual ends), and more recently neuroscience. Like all discourses, the ways of thinking and the practices that have been established within the broader topic of ‘educational research’ have been epiphenomena of a particular set of institutional conditions.

Organisations such as universities are characterised by formal rules and informal norms, in the form of rights, responsibilities, obligations and positions (North 1991; Lawson 1997) which are related to higher-order positioning, rights and obligations in society. The arrangement of rights and obligations sits on a natural/biological substrate, but the ontology of the relationship between nature and culture within the discourse on organisations and institutions has tended to focus on issues of epistemology and language, ignoring deeper biological foundations. With a few exceptions (e.g. Veblen (2017), Bataille (1991) and Lawson (1997)) accounts of institutional emergence rarely offer an explanation of the relationship beyond metaphorical allusion to natural selection and communication. Within evolutionary economics (Nelson 2009) or new institutionalism (Powell and DiMaggio 1991), the social dynamics of human language have supplanted the natural dynamics of cells. The characterisation of natural phenomena as linguistic phenomena considers evolution as a metaphor for the dynamics of development in economic systems. The underpinning role of natural systems is not explored.

Ontologically common to all these approaches is the view that organisations maintain their viability within an environment of which they form part. The inter-dependence of the organisation and its environment can be described in many ways. One may, for example, talk of the ontological ‘domains’ (Bhaskar 2008; Archer 2008), or the inter-relations between (usually three) worlds (Popper 1979; Habermas 1991), but what unites all descriptions is that there is an ongoing process which connects nature with culture. Either explicitly or implicitly, each process is described as ‘evolutionary’, detailed descriptions of the mechanism which drives the evolution of institutions from nature are under-specified, and attempts to address this are rarely found in the educational discourse (with some exceptions, for example Morin (2001) or Simondon (2017)). What is missing is any attempt to *explain* the processes that unite nature with culture and to support it with empirical evidence.

There is no shortage of homologous processes which appear to recur between social life and the natural world. Like organisms surviving in a niche which they construct, social organisations such as universities reproduce and transform their environment by creating the conditions for particular kinds of activities that they can then undertake. Such activities serve the reproduction of the organisation for generations to come. From the environment, organisations gain resources with which they can carry out their operations: money and people. The variety which is absorbed in terms of people (both students and staff) creates the conditions for organisations to create order: they organise themselves into departments, functions, classes and so forth. Inquiry into such homologous processes between cells and society becomes more urgent when the process that threatens the viability of our social organisations begins with an attack on our cells.

Systems theory and cybernetics address issues of homologous processes, and within this literature, there are descriptions of hierarchical processes linking culture and nature. Attempts by Beer (1972), Bateson (1987), Ashby (1960), Maturana and Varela (1980) and Luhmann (1995) provide a starting point for thinking about how mind and nature work together. Bateson enumerated ‘criteria of mind’ as involving information (in the processing of difference), energy (in order to feed the process) and circular organisation producing homeostasis, which in turn drive recurrent hierarchies of complex development (Bateson 1980).

Following Bateson, one might ask what effect an environmental disruption has on these hierarchical processes from cells to social organisations. From this question, missing elements in his description can be determined: most notably, the lack of account for the drive for continual development, or the critical role that the history of the environment/organism plays in determining evolution. Environmental disruption such as a disease presents an opportunity to empirically explore evidence to demonstrate the effects of stress at a cellular level, and whether this knowledge can help shape the future direction of educational organisations and learning.

### Recurrent Parameters of Organisation from Cells to Universities

Torday’s First Principles of Physiology (Torday 2012) echoes Bateson, recurring at different levels of biological organisation. Critically, however, from an epigenetic perspective, Torday argues that the evolutionary history of the organism encompasses the evolutionary history of the cell and its environment. Whilst the specific instantiation of Torday’s Principles varies at different levels of organisation, their function does not. One set of parameters exist at a cellular level, another at the level of the organism, another at the level of consciousness and so on. Each of these levels is connected, and evidence from one level can be used to support theories and hypotheses at other levels in a similar way to which evidence from physics informs testable predictions in chemistry through the Periodic Table of Elements.

From a basic perspective, the recursions of organisation from cells to universities can be summarised:

1. At the cellular level
  - The energy gained by the cell for its growth is established through chemiosmosis.
  - The cell transmits information with its environment through proteins by which it creates a niche for the species.
  - The cell’s internal mechanisms create order against the prevailing entropy of the physical environment.

2 The organism
  - The organism gains energy from the environment through food and sunlight.
  - the organism produces behaviours and epigenetic marks by which it creates a niche for the species.
  - The organism creates order through its ontogeny.
  3. The human being
  - The person gains energy and information from their environment.
  - The person produces communications, artefacts, tools, artworks, social processes and institutions.
  - The person creates order in their consciousness and understanding of the world.
  4. The university
  - The university gains resources in the form of money, personnel and land.
  - The university produces epigenetic marks in its environment in the form of research outputs, courses and graduates that help it to survive in the future.
  - The university creates order in its structures and practices using technologies to organise itself into academic departments and specific functions (e.g. accounts, estates, data management, and marketing).

The basic function of the First Principles of Physiology serves both the ontogeny of the organism and the phylogeny of the species. The latter is served by processes of epigenesis, as the outward expressions of information at different levels of organisation are deposited in the environment to be absorbed by subsequent generations of organisms. Since epigenetic marks are produced by organisms surviving and adapting in one kind of environment, which may subsequently be read by organisms in a different environment, the epigenetic mechanism provides a means by which the inheritance of acquired characteristics is possible.

This last point is important because moments of environmental stress on any organism will have lasting effects, not only on the individual but also on the species. Biological evidence for this shows that to be subjected to extreme heat or to be starved of oxygen are all processes that fundamentally change the evolutionary trajectory of organisms. For example, the pre-history of cholesterol, which is fundamental to animate life, could only have arisen under the specific extreme stresses of heat and pressure at the origins of life (Deamer 2017). Under such conditions, and by extension under the conditions of Covid-19, at every level of natural organisation from cells up, boundaries are moved and things reorganise to survive in their own environments, from cells up.

To shed light on what is happening to learners, teachers and universities, therefore, entails a study of what happens to organisms under stress. Covid-19 kills by putting bodies under stress in such a way that the cell’s response, which is an evolutionarily determined release of cytokines, causes the collapse of functioning of the complex bodily organs (principally the lungs and the kidneys) that they comprise. From the cell’s perspective, there is a logic to this. Faced with a fundamental change to the cellular environment, the priority is to find a new level of stability in the transformed cellular environment in which the cells find themselves. This new level of stability has been shown to be a level of stability at a prior evolutionary stage (Gould and Vrba 1982). The disease instigates an evolutionary regression to an earlier, more homeostatically controllable state of the cell under the prevailing conditions.

We argue that universities can be seen to be doing something similar. By viewing the total environment of learning as culture rather than nature, where culture is seen even more narrowly as ‘economics’ and ‘markets’, organisations will naturally perceive their role as ‘delivering learning’ to that market. The commandeering of technology in the rapid ‘pivot’ to online learning to ensure learning continues to be delivered is a kind of endogenisation process, by which educational organisations defend their established boundaries and economic model. Yet, this economic model itself is also threatened by the virus. By viewing their environment as defined by relations with culture, rather than nature, educational organisations risk embracing solutions that lead to further instability and, most critically, to the delivery of learning in ways that violate the biology of learning and human thought.

### Technology and the Evolutionary Regression of Institutions of Learning

The university was already under stress before Covid-19. Social organisations have always defended themselves against environmental threats, and with an increasingly complex environment expressed in terms of politics, markets and technologies, the need for organisational self-defence has increased, with technology playing a dominant role in institutional survival. However, Covid-19 represents an environmental threat from nature, contrasting the cultural phenomena that educational organisations have typically defended themselves against.

Culturally, the principal economic means of survival of educational organisations has been the manipulation of scarcity (Illich 1971; Illich 2009). Entrance criteria, examinations and certificates were originally scarce partly because the resources for teaching—books, lecture theatres, laboratories and teachers—were scarce. The Internet brought an explosion of possibilities to redistribute and reorganise the resources of education. This increase in possibilities also brought an increase in uncertainty in the environment: how should institutions embrace the plethora of digital technologies, given that they undermined the certainties of practice in the traditional academy?

The typical response for educational organisations has been to maintain and reinforce their grip on the mechanisms to create scarcity: their certificates and practices. The environmental uncertainty produced by technology has been suppressed by commandeering technology to reinforce organisational structures: publisher’s interests are protected behind paywalls, university students are offered educational ‘platforms’ which mimic (badly) the Internet outside, professional bodies have aligned their processes of accreditation with universities and academic progression is increasingly beholden to metrics derived from journals which have similarly artificially defended their scarcity (even to the point of requiring payment of fees simply to submit a paper for review, despite much of the review labour being provided for free to the journal’s controlling organisation). As a result, the cost to society has risen (Ilva, Laitinen and Saarti 2017), alongside ever more intense developments in technology to disrupt education: taken together, this has represented an increasing threat that organisational actions are exacerbating. In the face of Covid-19, this has resulted in the ‘pivot online’ where lectures continue to be delivered but in videoconferencing educational platforms.

Behind all systemic crises—whether it is in a cell or a society—is the uncontrollable oscillation produced by positive feedback (Beer 1972). This was the situation the marketised university was in before Covid-19. Correspondingly, the disease represents an environmental instability in an organisational situation that was already unstable. To explore possible strategies for educational organisations to successfully adapt to online learning and the implications of digital technologies more broadly, we propose that the focus of attention must shift to understanding the organisation’s relation with nature, rather than its relations with culture.

## Part 2: Evolution, Learning and Institutions

The epigenetic approach reveals individual consciousness to be emergent from cellular dynamics, which themselves carry a memory of their original development. By extension, the consciousness that education addresses must be more all-embracing than a superficial ‘market for education’ (Menand 2010; Collini 2017). Separating nature from culture in education is to separate the biology of individual learning from the biology of organisational viability.

As Covid-19 has caused the pursuit of online strategies for ‘delivering learning’, there are widespread reports of the stresses that these changes have had on both teachers and learners (Morrish 2019; Zhang et al. 2020; Moawad 2020; Canady 2020). The shifting of teaching and learning from face-to-face to online has not only resulted in a situation that appears unattractive for teachers and learners, but which has ushered-in a concrete change to the epigenetic environment for everyone, including others in the household. Most obviously, in a more stressful environment online, there will be an associated generation of the stress hormone cortisol, which through epigenetic mechanisms will have knock-on developmental effects on others in the environment.

### The Parameters of the Online Pivot

In the rush to maintain existing institutional practices, most institutions have conducted lectures and assessments online. Whilst functionally this appears to be the equivalent to face-to-face practice, numerous reports suggest that it is inducing more stress on both students and staff. This suggests that the parameters of face-to-face delivery do not match directly to those of online delivery. One possibility is that this problem is related to the market logic followed by educational organisations. However, if individual consciousness is emergent from cellular dynamics, which themselves carry a memory of their original development, then the consciousness that education addresses must be more all-embracing than a ‘market for education’—and be more than the acquisition of skills for clearly defined ‘professional’ roles.

The market logic has led to an increasingly transactional approach to education. The needs of consciousness in reconciling itself with its fundamental cellular origins could not be met in the transactional processes of lectures and assessments alone. The campus has been a critical and largely uninspected ingredient in the transactionalising of education: the epigenetic environment of the coffee bar, shared housing and the pub provide opportunities for individuals to establish deep relationships which recognise their common biological heritage. The business model of education grew on the back of the opportunities the campus provided. On the one hand, the business model instrumentalises the processes of assessment and accreditation, ostensibly creating markets for skill and knowledge. Yet in its physical and social contexts that bring students and researchers together, the university also continues to offer meaningful social opportunities which compensate for the stresses induced by the educational machine.

With the transactional aspects of educational engagement being represented by the online learning platforms, there is little to compensate for the intellectual self-organising processes of consciousness which potentially seek fidelity to a common biological heritage. Education after Covid-19 has not arrived at a stable state. The disruption to practice means that it may be not only unviable, but threatens to become insolvent.

Avoiding such regression entails recognising how the epigenetics of education involve expressions of creativity, thought, writing, talking, experimenting and inquiring in a way which fits the epigenetic conditions of the home. If the transactional processes of assessment and certification provide little support for individual creative or thoughtful expression, then the epigenetic processes which sustain the organisations will be under threat. In short, if the campus was the locus of epigenesis in face-to-face learning by virtue of the opportunities for biological connection, then the parameters of educational organisation must shift if the operation is to move online.

### Epigenetics and the Life of the Mind

Thought is a manifestation of complex biology. As such, thought embraces biological history in its ongoing development. From this perspective, thinking itself is an activity to reconcile complex biology with its origins in a similar way to that of simpler organisms, through processes of gathering energy, creating order and generating information. Thinking can also be seen as a process of being energised by difference in the environment, bringing order through conceptualisation and generating information in the form of utterances, writings and media.

Overlaid on this is the epigenesis and development of the organisational conditions within which biologically grounded thought can develop. Deep thought, and particularly the connections established through thinking deeply together, is both an individual journey into biological history, and a recognition of a shared biological heritage. Covid-19 provides an opportunity for embracing this journey, since its biological effects raise fundamental questions about the organisation of nature and consciousness.

In biological terms, the campus represents a ‘niche’ in which the practices associated with the university have a home. The parameters of stability in thought and language within the face-to-face institution and those of the online environment are necessarily different, because the niche is different. The market in education was predicated on a separation between nature and culture, such that the campus became an essential constituent of the educational enterprise, in a homologous way to which the cell constructed its first niche (Torday 2016). It represented the natural environment of university, whilst the transactional operation of education only recognised the cultural and economic environment. A side effect of this was that shallow thought and strategic learning could be acceptable and viable within an academy secure in its campus ‘niche’. However, as long as they continue to seek to maintain their existing structures and practices, our educational organisations cannot adapt to create the conditions to foster deep thought in a very different niche.

Mass education at a distance through an electronic medium provides a powerful means of developing a biologically grounded collective inquiry. Such a situation has existed before in the monastic pre-history of universities. Writing was the principal medium by which monks came to know each other, frequently exchanging texts and references with one another between the monasteries of Europe (Willinsky 2017). With the variety of today’s means of communication, opportunities for thinking together extend beyond text to, for example, synchronous videoconferencing, shared editing environments and self-publishing.

In the post-Covid-19 educational environment, perception of the passing of time and the sense of stress appear to reflect work on the experience of creative flow activities (Bruya 2010; Wright, Sadlo and Stew 2006; Rettie 2001). During the online pivot, experiences range from the ‘interminable’ Zoom meetings, lectures and overly long presentations, to the rapid passing of time in extended intellectual discussions in tutorials, research groups or playing online games socially. In inquiring about the epigenetics involved in such differences, stressful experiences will produce different hormonal responses to those experiences that engage deep levels of consciousness. More to the point, as phenomenologists like Schutz (1972) indicate, the intersubjectivity of deep levels of personal engagement indicates a fundamental ‘tuning-in’ to what he calls the ‘inner world’ (and by extension, the inner biology) of each other.

Online forums and mailing lists within scientific communities provide further evidence of the extent to which intersubjective engagement and deep thinking and expression of ideas does not demand face-to-face engagement, but can be satisfied with the simplest of media such as email. Yet this deep level of thought produces the same flow reaction, and consequently similar epigenetic marks, as those typically associated with face-to-face communication.

Recognising the biological significance of deep intellectual engagement online represents a distinct challenge for organisations. Whilst the transactional model of education that is driven by technology will dominate the conduct of online engagement, the technology itself can be used to facilitate deeper and more personal connection which is more in tune with the ontogeny of individual biology. The educational challenge is to find ways of reconciling biological history and identify its shared heritage with its environment. We propose that depth of thought is a parameter in the change of medium or ‘niche’ from face-to-face education to online and consideration of this parameter enables insight into transforming not only online education but also campus-based learning in the future.

### The University and Technology

The marketised university required a campus to increase the variety of human encounters (and hence its economic income) such that impersonal transactions of learning could be counterbalanced by deeper communicative and organisational processes within the rich variety of settings presented by the campus. However, an online organisation must function within very different parameters in order to satisfy biological epigenetic requirements. Without these compensations for the transactional aspects of learning, the shallowness of the transactional process is arguably insufficient to maintain the relationship between students and the university. However, if the epigenetics of the organisational engagement are understood as the relationship between organism and niche, then the parameters of engagement can be shifted creating the conditions for a different kind of niche construction.

The energy gained by an individual to grow intellectually has to be gained from the online environment and the depth of inquiry. It can only do this by embracing the totality of the Internet, the personal situation, the ‘home’ and the local environment, and not simply that part of the Internet which is marked as being ‘the course’. The epigenetics of the individual must similarly occur around use of technology, since the epigenetic marks produced by the individual will sustain both the individual and their peers, and their successors. In order to be able to maintain a sustainable niche that both supports meaningful individual adaptation and creates the conditions for the effective adaptation of the species, a deeper inner understanding is required that draws on cellular history.

But what organisational changes must an institution of learning make to sustain learning in an online environment? How is it to gain sufficient resource from its environment to maintain itself? How is to create order among its activities? How is it to generate information in its environment, and information of what sort?

At present, the operations of the university include the generation of courses and certificates, whilst its internal ordering processes concern the curriculum, timetables and exams, and its external information involves publications and other research outputs and marketing operations. Moving things online means that the potential for asynchronous engagement obviates the timetable, freedom of inquiry obviates the curriculum and creative expression of knowledge obviates exams. Moreover, because the online environment is global, the opportunities for harnessing networks of scholars from institutions across the world can be easily realised. In place of courses and certificates is a network of interesting people to have deep discussions with, and research outputs can take an increasingly varied form exploiting the full gamut of media available for expression. But such changes require a transformation of prevailing institutional conditions which will need to be situated on a solid empirical foundation.

Whilst it is beyond the scope of this paper to detail the specifics of potential new studies, mention may be made of ways of extending existing areas of research in education and society where empirical studies of the environment have been related to communication practices and phenomenological inquiries. These studies can take many forms, for example extending existing work monitoring epigenetic marks in the environment and situating these against communication behaviour, learning development (Bjorklund 2006; Youdell 2018; Pickersgill 2020) and physiological signals (data which is now easy to extract with fitness trackers). At a broader socio-economic level, extending work on communication dynamics and innovation systems (Leydesdorff 2006) arising among universities, government and industry, with additional data from epigenetic monitoring and physiological signals, can enrich understanding of the relationship between educational processes and their epigenetic context and socioeconomic development. This latter work is particularly interesting because its use of information-theoretical models provides the means whereby dynamics between different levels of organisation (for example, economic activity, communications, physiology) can be inter-related in a whole-system approach. Most pertinent the spirit of C.P. Snow articulated in this paper is the application of such empirical techniques to the comparative study of different kinds of learning activity—from creative and artistic work to work with logic, technology or mathematics, physical activity or spiritual practice. Such empirical studies can take many forms, with opportunities for modelling through simulation and prediction providing new approaches to social policy where social outcomes can be situated against more detailed specifications of the environment of learning.

## Critical Reflection: Objections to Our Approach

Like any new ‘grand narrative’ concerning education, the current buzz around epigenetics is likely to evoke suspicion. These concerns range from worries about the apparent ‘fadism’ of epigenetics itself, through to concerns about the reduction of complex social phenomena to biological mechanisms, and furthermore to obfuscatory misappropriation of scientific terminology. In this section, we want to address these concerns directly, particularly with respect to:

1. concerns about the reliability of epigenentics itself, and the range of work which calls itself ‘epigenetic’;
2. concerns about a reduction of educational phenomena to biology or physics;
3. concerns surrounding the deployment of scientific concepts to explain social phenomena to create an ‘aura’ of acceptability.

With regard to the first concern, whilst it is true that the word ‘epigenetic’ lacks clear definition (Meloni and Testa 2014), empirical practices involving the explicit manipulation of epigenetic marks to control organic development now represent a large portion of mainstream biology and physiology research and development. Epigenetic techniques of DNA methylation (the inhibition of gene transcription) are widespread in agriculture and medicine and their efficacy is beyond doubt (Zhang, Lang and Zhu 2018; Das and Singal 2004; Eckschlager et al. 2017). The relationship between epigenetic marks detected in the sperm of obese men has been studied for changes produced by bariatric surgery (Donkin et al. 2016), which clearly suggests the inheritance of acquired characteristics through epigenetics. Epigenetics has also been successful in producing stem cells from mature cells (what are called ‘induced pluripotent stem cells’) (Scudellari 2016), although scaling-up the production of stem cells has proved more challenging. Our deeper claim for the connection to consciousness and learning extends this work, on the basis of the evolutionary evidence as we have discussed. We would emphasise that the empirical basis for theoretical development is of primary importance. More extreme claims have been made for ‘epigenetic supplements’, or defences of pseudoscientific theories such as homoeopathy (Kanherkar et al. 2017), but here solid empirical evidence is to-date highly questionable.

With regard to the second concern, the charge of biological reductionism is often invoked as a criticism of approaches to social or psychological phenomena which overlook concerns about specificity in social organisation (Sayer 2010). The essence of the critique lies in a fear that reduction causes blindness to the stratification of social mechanisms (a problem that Bhaskar (1993) calls ‘ontological monovalence’), and which may in turn lead to social aporia. A good example is the reduction of social progress to results from intelligence tests or to algorithmic processes. These are of course very valid concerns.

‘Reduction’ as a process of distilling complex phenomena to some core generative mechanism lies at the heart of all scientific inquiry. As Sayer (2010) notes, problems with reduction lie not in this distillation process itself, but in its effects. The distillation process effected by Newton or Einstein produced major advances for civilisation, which although not free from aporia, resulted in a significant increase in the variety of human life. Whilst simplistic ‘reduction’ in terms of race, gender, sexuality or political sympathy all have been shown to produce a catastrophic reduction in the variety of human life and collapse of civilisation, scientific abstraction is often an intellectual move that counters this, creating the conditions for social progress.

This problem of reductionism is closely related to the third concern we wish to address. In the social sciences, the distillation of complex social phenomena to under-specified scientific terminology becoming a vehicle for obfuscatory work which was highlighted in the scandal of a fake paper written by Alain Sokal and documented in his book *Intellectual impostures* and his subsequent reflections (Sokal and Bricmont  1999; Sokal 2009). Whilst it has been argued that Sokal overstated his case (which was against a range of significant figures in philosophy and sociology including Lacan, Latour and Deleuze), the problem of appropriation of scientific terminology in the social sciences continues despite Sokal’s criticism (the ubiquity of the sociological use of the term ‘entanglement’ is a good example).

Obfuscation is not only a problem of current science (for example, quantum mechanics) but also a problem of older science which underpins commonly accepted contemporary social or psychological theory. This is the case with educational theory, where the roots of Piagetian epistemology lie in the biology of the 1920s. Whilst it creates a disruption to the current educational discourse to return to the biological roots of theory, re-evaluation of those roots in the light of current science is necessary for the discourse to develop. Avoiding the fossilisation of theory, defended within established institutional structures, discursive boundaries and methodological practices are precisely our motivation for this paper. Highlighting the importance of epigenetics in bringing to light the dynamic relation between environment, cellular development, consciousness and social structures is a way of shining a light back onto the scientific roots of our sociological and psychological assumptions about learning and education.

## Conclusion

This paper has argued that one effect of Covid-19 on education has been to highlight the relationship between nature and culture. By articulating an approach to evolutionary biology that emphasises the common thread that unites cellular development with the development of thought, we have suggested reasons why education is particularly threatened by Covid-19, and what might be done about it. We have presented evolutionary biology and epigenetics as a foundation for an argument for reconfiguring the parameters of learning and educational organisation.

The ‘online pivot’ in the face of the virus raises questions about the parameters with which universities and individuals can be viable in their ongoing processes of learning. We have argued that the marketised university saw its environment as culture, and ignored the role that nature played, which was hidden in the role of the campus. Without the campus in the online environment, consciousness and thought must be re-inspected as fundamental parameters in the establishment of viable relationships of learning in a distance medium, and in the creation of conditions for the ongoing survival of the organisational context for learning.
